# Management of Antidepressant-Induced Sexual Dysfunction: A Literature Review

**DOI:** 10.7759/cureus.90170

**Published:** 2025-08-15

**Authors:** Faith D Tran, Gregory H Bichai, Zachary Kravetz, Zina Meriden

**Affiliations:** 1 Psychiatry, Florida Atlantic University Charles E. Schmidt College of Medicine, Boca Raton, USA; 2 Pediatric Medicine, Yale New Haven Hospital, New Haven, USA; 3 Psychiatry, Memorial Regional Hospital, Hollywood, USA

**Keywords:** antidepressant-induced sexual side effects, antidepressant sexual side effects, antidepressant side effects, psychotropic-related sexual side effects, psychotropic side effects

## Abstract

Although selective serotonin reuptake inhibitors (SSRIs) are the mainstay of treatment for various psychiatric conditions, including but not limited to major depressive disorder, generalized anxiety disorder, obsessive-compulsive disorder, and bulimia nervosa, sexual side effects from these medications are common. These side effects include decreased libido, difficulty with arousal and erection, and delayed or absent orgasm, which can be troubling for many patients and impact adherence to treatment and quality of life. This is an important distinction from decreased libido that can be seen in the natural course of depression; the sexual dysfunction discussed in this paper is due to an adverse effect of SSRIs.

The goal of this narrative review is to explore the effectiveness of switching antidepressant therapy or adding adjunctive medications or herbal supplements in alleviating antidepressant-induced sexual dysfunction (AISD).

An electronic literature search was performed on PubMed to identify full-text, English-language articles that discussed antidepressant-induced sexual dysfunction (AISD). Selected articles were published between 1996 and 2023.

Replacing one antidepressant class for another with a more favorable side effect profile (e.g., mirtazapine, nefazodone, and vortioxetine) may be effective for some patients, especially if the initial antidepressive treatment was not sufficient in controlling the symptoms caused by the primary mental illness. Several adjunctive medications and supplements also show promise in the management of AISD, including bupropion and saffron (*Crocus sativus*). Findings surrounding other adjunctive treatments are promising, though more robust studies are needed. Evidence for switching to vilazodone to treat AISD is conflicting, as is data surrounding the effect of Ginkgo biloba on AISD. Data surrounding AISD management is increasing, but remains relatively scarce. Further studies are necessary to understand AISD’s pathophysiology and current interventions. Development and use of other drugs, supplements, and nonpharmacological agents should be explored in this patient population to broaden effective management options for AISD.

## Introduction and background

Methods

A literature search was performed in Pubmed® (National Library of Medicine) through September 2023 with the following search terms: “antidepressant-induced sexual dysfunction,” “antidepressant sexual side effects,” “mechanism," and "treatment of antidepressant-induced sexual dysfunction.” Full-text, English-language articles that covered the subject of sexual dysfunction secondary to antidepressant treatment were reviewed. Select articles that do not specifically cover this topic were referenced if needed for the explanation of an agent’s mechanism of action. All other articles were excluded from this narrative review. All articles included in the review were published between the years 1996 and 2023. The references of several papers were also reviewed for additional relevant publications.

Introduction: SSRIs and the sexual dysfunction they cause

Sexual dysfunction is a condition common in both men and women that can be caused by variations in hormone levels, medical illnesses, medications, or attitude towards sex/intimate partner [1]. Symptoms of sexual dysfunction include decreased libido, difficulty with arousal (resulting in vaginal dryness or erectile dysfunction), and delayed or absent orgasm [2]. While depression itself is often associated with sexual dysfunction (typically in the form of decreased libido), the use of an antidepressant may ironically exacerbate this by causing more sexual dysfunction (typically in difficulty with arousal and anorgasmia) [3]. Sexual problems (such as impotence, ejaculatory dysfunction, dyspareunia, vaginismus, anorgasmia) were found in 26-31.8% of normal subjects, 45% of non-treated depressed patients, and 62-63% of treated depressed patients. Adverse effects of sexual dysfunction can affect adherence to medications [4, 5].

Selective serotonin reuptake inhibitors (SSRIs) such as citalopram, fluoxetine, escitalopram, paroxetine, and sertraline are the most common antidepressant class linked to sexual dysfunction, primarily difficulties with orgasm or ejaculation [6, 7]. Some studies suggest that as many as 80% of patients taking SSRIs experience some form of sexual dysfunction [8]. Several mechanisms underlying such dysfunction have been posited. One theory of sexual dysfunction secondary to SSRI use involves variation in genes involved with the serotonin system, such as *HTRA2*, *SLA6A4*, *BDNF*, and the glutamate system, including *GRIK2*, *GIRa3*, and *GRIA1* [9, 10]. Dopamine, norepinephrine, gamma-aminobutyric acid, acetylcholine, nitric oxide, and oxytocin are other neurotransmitters that modulate the various stages of sexual function. Increased serotonin throughout the body may interfere with testosterone and neurotransmitters such as dopamine [10, 11]. SSRIs, monoamine oxidase inhibitors (MAOIs), and tricyclic antidepressants can increase levels of prolactin, adversely impacting orgasm and arousal [12-14].

The goal of this narrative review is to explore the effectiveness of switching antidepressant therapy or adding adjunctive medications or herbal supplements in alleviating antidepressant-induced sexual dysfunction (AISD). Switching the patient from SSRI therapy to another medication can reverse the sexual side effects that the SSRI treatment caused. There are also prescription medications and herbal supplements that have been studied with possible roles in mitigating AISD when used as adjunctive therapy. The purpose of this literature review is to explore the available data and highlight these agents, looking also at their mechanisms of action and effects on sexual function. Table 1 summarizes the mechanisms of action of all the medications and herbal supplements discussed in this review.

**Table 1:** Mechanism of Action for Each Drug or Herbal Supplement 5-HT1: 5-hydroxytryptamine receptor 1 5-HT1A: 5-hydroxytryptamine receptor 1A 5-HT1B: 5-hydroxytryptamine receptor 1B 5-HT1D: 5-hydroxytryptamine receptor 1D 5-HT2: 5-hydroxytryptamine receptor 2 5-HT3: 5-hydroxytryptamine receptor 3 5-HT7: 5-hydroxytryptamine receptor 7 SERT: Serotonin transporter α-2: Alpha-2 adrenergic receptor H1: Histamine H1 receptor

​​Medication or Herbal Supplement	​Mechanism of Action
​Bupropion	​Dopamine and norepinephrine reuptake inhibitor
​Mirtazapine	​5-HT2, 5-HT3, α-2, and H1 receptors antagonist (thus enhances the release of norepinephrine and serotonin to bind the 5-HT1 receptors)
​Moclobemide	​Reversible inhibitor of the monoamine oxidase A enzyme
​Nefazodone	​5-HT2 blocker
​Vilazodone	​5-HT1A partial agonist, selective serotonin reuptake inhibitor (via SERT inhibition)
​Vortioxetine	​5-HT3, 5-HT7, and 5-HT1D receptor antagonist. 5-HT1A receptor agonist, 5-HT1B receptor partial agonist, and selective serotonin reuptake inhibitor (via SERT inhibition)
​Ginkgo biloba	​Increases blood flow to the brain and genitalia, improves erectile dysfunction, induces serotonin and norepinephrine receptors in the brain, and increases nitric oxide synthesis.
​Maca root (Lepidium meyenii)	​Reduces fatigue by improving the activity of glutathione peroxidase and creatine kinase
​Mianserin	​5-HT2 agonist, partial 5-HT1 and 5-HT3 agonist, α-2 antagonist, and potential H1 antagonist
​Saffron (Crocus sativus)	​Inhibits serotonin reuptake and increases levels of nitric oxide in the body
​SAMe (S – Adenosyl – L – Methionine)	​Universal methyl donor
​Ristela (French maritime pine bark extract, L-arginine, L-citrulline, and specialized rose hip extract)	​Increases nitric oxide production and blood flow, and also increases oxygen and nutrient delivery to reproductive organs
​Rosa damascena	​Antagonizes 5-HT2 and 5-HT3 postsynaptic receptors, increases dopamine and norepinephrine release in substantia nigra, and disinhibits nitric oxide synthase
​Tribulus terrestris	​Increases levels of testosterone, increases blood flow in the clitoral artery

This article was previously presented as a platform presentation at the 2024 Florida Atlantic University Research and Scholarship Day on April 16, 2024.

## Review

Switching antidepressant medication

A common mitigation strategy for intolerable adverse effects of medication includes switching to an agent with a different side effect profile. The same is true for antidepressants and their adverse effect on sexual dysfunction. Below, we explore different agents that can have a better side effect profile in the context of AISD.

Mirtazapine

Mirtazapine has been shown to be an effective antidepressant for patients suffering from AISD. Mirtazapine antagonizes the 5-HT2, 5-HT3, α-2, and H1 receptors, enhancing release of norepinephrine and serotonin, which then bind to 5-HT1 receptors. This results in an antidepressant response with a reduced sexual side effect profile [15, 16]. In an open-label six-week study by Gelenberg et al., 19 patients already on an antidepressant and with AISD were subsequently switched to mirtazapine, with doses ranging from 7.5-45 mg. The Arizona Sexual Experiences Scale (ASEX) was used to assess sexual function, with patients with ASEX scores < 19 being named “responders.” Depression severity was measured using the Hamilton Depression Rating Scale (HAM-D). While responders and non-responders had similar HAM-D scores, the responders had reduced ASEX scores in multiple domains other than orgasm satisfaction, with mean scores dropping from 22 ± 22 to 16 ± 6 (*p *< 0.001). While encouraging, the small study sample, lack of blinding, and lack of a control group are limitations of the study [15].

Moclobemide

Moclobemide, a reversible inhibitor of the monoamine oxidase A enzyme, may have more favorable side effects relative to other antidepressant classes and may help reduce sexual dysfunction [17, 18]. In an open-label, prospective, and observational study examining 107 patients with major depressive disorder on either moclobemide, sertraline, paroxetine, or venlafaxine. The authors looked at the incidence of sexual dysfunction among different antidepressants; they did not evaluate switching or adjunctive use. A 10-item Sexual Function Questionnaire (SFQ) and a 17-item HAM-D to assess sexual function and depression severity, respectively, were administered prior to and after eight or 14 weeks. All four antidepressants were equally effective at reducing depression. Those on moclobemide and venlafaxine experienced less sexual dysfunction compared to those on paroxetine or sertraline (χ² = 8.51, df = 1, *p* < 0.004 by 2x2 chi-square analysis). Moclobemide showed less difficulty with arousal and reaching orgasm in women compared to the other three drugs. Men were more likely than women to experience impairment of drive or desire in a drug-related fashion. One limitation regarding the use of moclobemide is that it is unavailable in the US [19].

Nefazodone

Nefazodone is a serotonin antagonist and reuptake inhibitor, blocking 5-HT2A and 5-HT2C, that also increases epinephrine and blocks serotonin reuptake less than SSRIs. It may be used in lieu of SSRIs associated with sexual dysfunction [20]. One double-blind, eight-week study examined 72 patients with major depressive disorder with sertraline-induced sexual dysfunction. Patients were given either sertraline 100 mg (n = 33) or nefazodone 400 mg daily (n = 39) after a one-week wash-out period and 7-10-day phase of receiving placebo only. Three times as many (76%) of patients in the sertraline group had difficulty with ejaculation as compared to nefazodone (26%) (*p *< 0.001) [21]. One cross-sectional, observational study conducted among 1101 U.S. primary care clinics (1763 men and 4534 women) showed that SSRIs, mirtazapine, and extended release venlafaxine had a 36-43% association with sexual dysfunction symptoms as compared to 28% for nefazodone [22]. A multicenter, prospective, open-label study of 1022 patients from Spain highlighted that the incidence of sexual dysfunction due to nefazodone was 8%, compared to an incidence of 57-73% due to SSRIs such as fluoxetine, sertraline, fluvoxamine, paroxetine, and citalopram [17].

Vilazodone

Vilazodone, an SSRI which works at the level at the serotonin transporter (SERT) and at both presynaptic and postsynaptic 5-HT1A receptors, has been touted as having fewer sexual side effects compared to other SSRIs; however, evidence for its role in mitigating AISD is conflicting. It is the 5-HT1A partial agonism that accounts for the lower likelihood of vilazodone causing sexual dysfunction. In a randomized, double-blind, parallel-group, phase I study over five weeks, a total of 170 participants were given 20 or 40 mg of vilazodone, 20 mg of paroxetine, or placebo daily [23]. Sexual function was tracked using the Change in Sexual Functioning Questionnaire (CSFQ). There were two groups within the active treatment group. The first was the modified intent-to-treat-I group (mITT-I), who had detectable drug blood concentrations during all post-baseline study visits. The second was the modified intent-to-treat-II group (mITT-II), who had detectable drug concentrations in their blood during any (but not necessarily all) post-baseline study visits. The decrease in overall CSFQ score was most pronounced in men in the m-ITT-II group (*p *< 0.05). Those on vilazodone 20 mg had a statistically significant reduction in CSFQ score compared to paroxetine in both groups (*p *< 0.05) [24]. Another study analyzing data from three Phase III studies showed results which contradict the previous study. Men and women with major depressive disorder and sexual dysfunction were given either vilazodone (titrated up to 40 mg daily) or placebo over eight weeks. In this case, CSFQ score improved in the placebo, not the vilazodone group [25].

Vortioxetine

Vortioxetine is an antagonist of the 5-HT3, 5-HT7, and 5-HT1D receptors, an agonist of the 5-HT1A receptors, a partial agonist of the 5-HT1B receptors, and lastly, an inhibitor of SERT [26]. The antagonism of the 5-HT3 and 5-HT7 receptors ultimately leads to loss of inhibition or reduced inhibitory tone, resulting in an increase in dopamine and norepinephrine. In a phase 4, multicenter, double-blind, placebo-controlled, 4-arm, fixed-dose, and head-to-head study, 361 patients were either given vortioxetine (10 mg or 20 mg), paroxetine 20 mg, or placebo for five weeks, with the chief outcome measure being assessed using the Changes in Sexual Functioning Questionnaire Short Form (CSFQ-14) score at five weeks with vortioxetine compared to paroxetine. Vortioxetine 10 mg caused fewer sexual side effects compared to paroxetine, which was statistically significant, with a mean difference of +2.74 points (*p* = 0.009). Vortioxetine 20 mg also had fewer sexual side effects compared to paroxetine, but the effect was not statistically significant [27]. In another study, 447 initial patients who were being given and effectively treated to citalopram, paroxetine, or sertraline were randomized to either vortioxetine 10 mg or 20 mg vs. escitalopram 10 mg or 20 mg with sexual function being assessed using the CSFQ-14 at eight weeks and response to antidepressant measured using the Montgomery-Asberg Depression Rating Scale (MADRS), Profile of Mood States brief form (POMS-brief), and Clinical Global Impressions Scale (CGI). Use of vortioxetine was associated with statistically significant improvement in the CSFQ-14 score at eight weeks (8.8 ± 0.64, mean ± standard error) relative to escitalopram (6.6 ± 0.64; *p* = 0.013), especially in desire, arousal, and orgasm [28]. Vortioxetine and escitalopram were equally as effective as antidepressants.

Adjunctive therapy: medications

Though switching medications is an option, this may not be favorable in patients who are otherwise tolerating their antidepressant therapy well. Different adjunctive medications can be considered and added to a patient's medication regimen to help alleviate AISD.

Bupropion

Bupropion is an atypical antidepressant that inhibits dopamine and norepinephrine reuptake and does not significantly affect serotonin levels. As such, it is associated with fewer sexual side effects compared to SSRIs. It may be effective on its own for those who have experienced SSRI-induced sexual dysfunction or as a treatment adjunct. In a randomized, double blind, fixed-dose study of 218 women with SSRI dysfunction, patients received either bupropion SR 150 mg twice daily or placebo for 12 weeks. The primary efficacy outcome measure was sexual function as assessed by the Female Sexual Function Index (FSFI). The mean FSFI score was 25.9 vs. only 17.2 in the placebo group (*p* = 0.001), with higher scores in desire, arousal, lubrication, orgasm, and satisfaction with sexual function [29]. In one placebo-controlled, double-blind study of 55 subjects, 150 mg of bupropion SR given twice daily was shown to increase sexual desire and frequency of sexual activity compared to placebo at four weeks (*p* = 0.024) [30]. In another randomized, double-blind, placebo-controlled study, 42 participants (37 women, five men with a mean age of 39 ± 5.7 years) with AISD were given 150 mg of bupropion SR twice daily [31]. Sexual function and depression were then measured by the CSFQ and HAM-D score, respectively. Results showed a statistically significant increase in sexual desire/frequency (*p* = 0.024) but no statistical significance difference in the overall CSFQ score. In another clinical trial, 18 participants (mean age 40 ± 12.5 years) experiencing unacceptable sexual dysfunction from venlafaxine extended release, paroxetine, or fluoxetine received adjunctive 150 mg of bupropion SR once a day for eight weeks. There was a significant decrease in delayed orgasm (*p* = 0.02) when bupropion sustained release was combined with venlafaxine, paroxetine, or fluoxetine in women [32].

Mianserin

Mianserin is a tetracyclic antidepressant, a strong 5-HT2 agonist and, to some extent, a 5-HT1 and 5-HT3 agonist, α-2 antagonist, and potential H1 antagonist [33]. Mianserin has been investigated as a potential treatment for various conditions, including depression and sexual dysfunction. Mianserin may have a role in successfully improving sexual dysfunction due to SSRI use in patients with traumatic brain injury [33]. One limitation is its lack of availability in the US. One clinical study examined 17 participants with traumatic brain injury (16 men and one woman, mean age of 40.8 years) suffering from AISD due to fluoxetine (n = 7), paroxetine (n = 6), citalopram (n = 2), or sertraline (n = 2). Ten of these patients received 7.5 mg of mianserin while the rest received 15 mg. Most were followed for three months (three participants were followed for six months instead to evaluate for any side effects). Their responses to mianserin supplementation were rated using the Clinical Global Impression Scale for Improvement (CGI-I) scale, with lower scores indicating greater improvement in sexual function. At the two-month mark, 10 patients reported a CGI-I score of 1, which refers to “very much improved” sexual dysfunction with no symptoms surrounding sexual activity. Delayed ejaculation was the fastest adverse effect to respond to mianserin. All 10 participants who reported “very much improved” in sexual function (CGI-I = 1) with mianserin had been suffering from delayed ejaculation before treatment [33].

SAMe (S-Adenosyl-L-Methionine)

SAMe is a chemical derived from the amino acid methionine. SAMe has shown promise in treating various medical conditions, including hepatic diseases, Alzheimer’s disease (AD), and major depressive disorder (MDD) [34, 35]. A single-center, six-week, randomized, double blind study looked at 58 patients taking unspecified SSRIs or selective norepinephrine reuptake inhibitors (SNRIs) with adjunctive SAMe therapy (400 mg twice daily for two weeks, followed by 800 mg twice daily) relative to placebo. Sexual function and depressive symptoms were then measured using the Massachusetts General Hospital-Sexual Functioning Questionnaire (MGH-SFQ) and HAM-D scores, respectively. Adjunctive SAMe treatments had a positive impact on male arousal (3.0 ± 1.2; *p* = 0.0012) and erectile dysfunction (3.1 ± 1.3; *p* = 0.01) independent of depression scores [36].

Adjunctive therapy: herbal supplements

Though switching medications is an option, this may not be favorable in patients who are otherwise tolerating their antidepressant therapy well. Adjunctive therapy with another medication can also be considered, but many patients are skeptical about adding additional medication. Different adjunctive herbal supplements have been explored in the literature to see if they can help alleviate AISD. Learning more about these herbal supplements can also help us better understand AISD and medications that can be used to alleviate it.

Tribulus terrestris

*Tribulus terrestris* is an herbal plant that may improve clitoral artery blood flow in post-menopausal women and increase testosterone levels in both pre- and post-menopausal women [37]. In a randomized, double-blind, placebo-controlled clinical trial study of over 67 women with hypoactive sexual desire disorder, participants in the treatment group received 7.5 mL of *Tribulus terrestris* twice a day for four weeks beginning the day after completing menses. Sexual function was assessed by the FSFI. Results showed improved scores for sexual desire (*p *< 0.001), arousal (*p* = 0.037), lubrication (*p *< 0.001), orgasm (*p *< 0.001), satisfaction (*p *<0.001), pain (*p* = 0.041), and overall combined FSFI scores (*p* = 0.040) [38]. In a randomized, double-blind, placebo-controlled trial, 41 women on sertraline with MDD and AISD were given two Aphrodite tablets daily (each tablet consisted of 40 mg of *Tribulus terrestris*) for eight weeks. At the four and eight-week marks, participants’ sexual function, depressive symptoms, anxiety, and sleep were assessed using FSFI, Beck Depression Inventory (BDI), Beck Anxiety Inventory (BAI), and Pittsburgh Sleep Quality Index (PSQI), respectively. Results showed statistically significant improvements in the Aphrodite group in all four indices (*p* = 0.001) [39].

Saffron

Saffron (*Crocus sativus*) is a plant that inhibits serotonin reuptake and can treat mild to moderate depression [40]. It also increases levels of nitric oxide in the body, making it easier to achieve an erection [10]. In a randomized, double-blind, placebo-controlled study, 30 male participants who experienced fluoxetine-induced sexual dysfunction (40 mg) received an adjunctive therapy of 15 mg of saffron twice a day for four weeks. Results showed a significant improvement in the saffron group’s self-report of erectile function (*p *< 0.001), scored using the International Index of Erectile Function scale (IIEF; score < 25 indicates erectile dysfunction). Sixty percent of patients in the saffron group had improvements in their IIEF scores by week four (*p *= 0.005) [41]. Another four-week, randomized, double-blind, placebo-controlled study on the use of saffron in 38 female participants with fluoxetine-induced sexual dysfunction was conducted. Sexual function was assessed using the FSFI. The mean age in the saffron group (15 mg twice daily) was 34.7 ± 4.7 years, and the mean age in the placebo group was 36.0 ± 6.1 years. Results at the end of the trial showed a statistically significant improvement in the total FSFI score (*p *< 0.001) as well as absolute mean changes in domain subscores relating to sexual arousal (0.49 ± 0.84; *p* = 0.028) lubrication (0.46 ± 1.11; *p* = 0.035), and pain (0.49 ± 0.73; *p* = 0.016), when compared to placebo [42].

Maca Root

Maca root (*Lepidium meyenii*) is a Peruvian root that is rich in fiber, amino acids, vitamin C, iron, and other vital nutrients. It has been used to treat sexual dysfunction, infertility, fatigue, inflammatory conditions, and depression [43]. In a 12-week, double-blind, placebo-controlled study, 45 pre-menopausal and post-menopausal women on an SSRI (unspecified), venlafaxine, or a tri/hetero cyclic antidepressant (unspecified) for four weeks prior to participating in the study were given three grams of maca root daily or placebo. Participants’ sexual function was then assessed using the MGH-SFQ and ASEX scores as primary endpoints [44]. A higher percentage of those in the maca group achieved improvement on the ASEX (total score ≤ 10: 9.5% for maca versus 4.8% for placebo) and MGH-SFQ (total score ≤ 8: 9.5% for maca versus 5.0% for placebo). While maca root shows promise in women suffering from antidepressant-induced sexual dysfunction, more studies should be conducted in those using various antidepressants, whether within the same class or not. More studies should also be conducted in men, as one 12-week study in men showed improvements in sperm concentration and motility with the addition of maca [45].

Ginkgo biloba

Ginkgo biloba (in particular, the EGb761 extract) comes from Asia’s maidenhair tree and can increase blood flow to the brain and genitalia, improve erectile dysfunction, activate serotonin and norepinephrine receptors in the brain, and increase nitric oxide synthesis [46]. In one open trial, 63 patients taking SSRIs (fluoxetine, sertraline, and paroxetine) or other antidepressants (nefazodone, bupropion, venlafaxine, phenelzine, Vivactil) for four weeks were given adjunctive Ginkgo biloba (40-60 mg twice daily, titrated up to 120 mg twice daily). Those receiving adjunctive Ginkgo biloba showed an 84% efficacy in alleviating sexual dysfunction secondary to antidepressant use. The response rate was lowest for fluoxetine (46%) and venlafaxine (69%), with women showing more success relative to men (91% versus 76%) [47]. There have been two studies with findings that conflict with the aforementioned open trial. In the first, which was a placebo-controlled double-blind study of 37 participants with AISD from fluoxetine or paroxetine, supplementation with Ginkgo biloba daily of 120 mg x in weeks 1 and 2, 160 mg in weeks 3 and 4, and 240 mg from weeks 5 through 8 caused only one statistically significant finding - satisfaction to orgasm at eight weeks. Otherwise, there was no statistical significance compared to placebo at weeks 2, 4, and 8 [46]. In the second, an open-label study of 22 patients with AISD, given 300 mg of Ginkgo biloba three times daily, only three had at least partial improvement. This study had several limitations, including a small sample size and a lack of a placebo control group [48].

Ristela

Ristela is a combination of French maritime pine bark extract, L-arginine, L-citrulline, and a specialized rose hip extract aimed to increase orgasm and sexual arousal in women. One open-labeled trial examined the effects of Ristela on the sexual function of 30 peri-menopausal and post-menopausal women on SSRIs, SNRIs, tricyclic antidepressants (TCAs), or atypical antidepressants (all unspecified). The treatment group received two tablets of Ristela daily for eight weeks, with FSFI scores checked at weeks 2, 4, and 8. Using Ristela alongside their antidepressant medication, at eight weeks, women reported increases in sexual arousal by 65%, orgasm by 61%, and desire by 55%, respectively. While arousal and orgasm improved with the concomitant use of Ristela and antidepressants, the effect on desire was not as strong [49].

Rosa damascena

*Rosa damascena* oil may improve sexual dysfunction secondary to SSRI use in men. It may exert its effects by antagonizing the 5-HT2 and 5-HT3 postsynaptic receptors and increasing norepinephrine and dopamine release in the substantia nigra [50]. One randomized eight-week double-blind study oversaw 60 male patients (mean age of 32 years) suffering from major depressive disorder and AISD. The patients were separated into two groups: a treatment group (given 17 mg of Citronellol of *Rosa damascena* oil) and a control group, while also continuing to take their antidepressant medications at doses they had taken for six weeks (duloxetine, escitalopram, venlafaxine, and sertraline). This study showed both significant improvements found in depressive symptoms (using the BDI) and sexual symptoms (using the BSFI) in the *Rosa damascena* group. By week 8, scores in sexual drive and overall satisfaction (*p* < 0.05), as well as erections, ejaculation, problem assessment, mean BSFI score, and BDI (*p* < 0.001) had improved [50].

Limitations

Common study limitations, especially in the herbal supplements, include but are not limited to a few number of studies, small sample size, short duration of treatment with the adjunctive therapy, variation in dosing amongst studies, limited generalizability, lack of multiple randomized control trials, and lack of a control group. Studies that cover the same adjunctive therapy also often did not have subjects on the same antidepressive treatment. Additionally, further study of these medications and supplements is required to observe their long-term effectiveness for the management of AISD. More conversation on the suspected mechanism of action for these adjunctive medications and adjunctive herbal supplements can help us understand AISD better in general. Despite barriers, there is promising data present, and future research will aid in our understanding of AISD, with the ultimate goal being to improve the quality of life for this patient population.

Limitations of this study also include the fact that the authors extracted articles from only one database in one language. This study also did not explore non-pharmacological interventions such as couples counseling or lifestyle changes.

## Conclusions

Antidepressants, especially SSRIs, play an integral role in the treatment of multiple mental illnesses, including but not limited to depressive disorders, anxiety disorders, and bulimia nervosa; however, a common adverse effect of antidepressants is sexual dysfunction (most commonly anorgasmia and difficulties with arousal). This side effect adversely impacts treatment adherence and overall quality of life. Patients who are being treated with antidepressants, especially SSRIs, should be screened for sexual dysfunction throughout their illness to determine what symptoms may be caused by the primary mental illness, and what symptoms could be caused by the medications we prescribe them. Prescribers should not rely on patients' self-reporting alone, as sexual dysfunction often is accompanied by embarrassment and shame. Interventions and options should be discussed with the patient in an open conversation. Screening/assessment tools such as the Changes in Sexual Functioning Questionnaire and Hamilton Depression Rating Scale can be given to patients before and after beginning SSRIs to trend symptoms of sexual dysfunction as well. The goal of this narrative review was to explore the effectiveness of switching antidepressant therapy or adding adjunctive medications or adjunctive herbal supplements in alleviating antidepressant-induced sexual dysfunction (AISD).

Switching antidepressants to another agent is a preferable strategy for many patients, especially if the initial agent did not show adequate resolution of symptoms caused by their primary mental illness. Medications such as mirtazapine, nefazodone, and vortioxetine are effective substitutes for antidepressants that disrupt sexual function. Moclobemide monotherapy did show less AISD compared to other SSRIs, but this study was observational, meaning moclobemide was not used as replacement therapy. Moclobemide is also not approved for use in the United States. Vilazodone did not have compelling evidence in being an alternative medication for patients with AISD. Adjunctive medication can be considered, especially if the antidepressant being used is showing effectiveness in alleviating the symptoms associated with a patient's primary mental illness. Bupropion shows strong evidence as an effective adjunctive treatment for AISD. It is often even used as monotherapy for many mental illnesses. S-Adenosyl-L-methionine has some compelling data, but there is a small number of robust studies. Mianserin also has some promising data available, but studies within the United States are limited. For adjunctive herbal supplements, saffron (*Crocus sativus*) and *Tribulus terrestris *both showed compelling evidence in being effective adjunctive treatments for the management of ASID. Maca root (*Lepidium meyenii*), Ginkgo biloba (in particular, the EGb761 extract), and Ristela (the combination of French maritime pine bark extract, L-arginine, L-citrulline, and a specialized rose hip extract) all were lacking in the number of robust randomized controlled trials, and the ones that were present showed varying results. Studies were also limited by small sample size.
